# Spontaneous closure of macular holes secondary to posterior uveitis: case series and a literature review

**DOI:** 10.1186/1869-5760-3-34

**Published:** 2013-02-11

**Authors:** Nicolas Bonnin, Pierre-Loïc Cornut, Florian Chaise, Elodie Labeille, Helene Janin Manificat, Audrey Feldman, Laurent Perard, Franck Bacin, Frédéric Chiambaretta, Carole Burillon

**Affiliations:** 1 Service d'Ophtalmologie, Pôle Médecine Interne-Ophtalmologie-ORL, CHU Clermont-Ferrand, Clermont-Ferrand, 63003, France; 2 Department of Ophthalmology, Hospices Civils de Lyon, University Hospital, Claude Bernard Lyon I University, Lyon, 69008, France; 3 Department of Internal Medicine, Edouard-Herriot Hospital, place d'Arsonval, Lyon, 69008, France

**Keywords:** Posterior uveitis, Macular hole, *Toxocara canis*, Sarcoidosis, Syphilis

## Abstract

The occurrence of a macular hole due to posterior uveitis is infrequently reported. We report the evolution of three cases of macular holes secondary to posterior segment inflammation. A complete inflammatory and infectious assessment found one case of toxocariasis, one of sarcoidosis, and one of syphilis. After medical etiological treatment, macular hole closure was rapidly obtained in all the cases and confirmed by spectral domain optic coherence tomography, with visual acuity improvement. Fibrous scarring developed in two cases, and foveal photoreceptor complex normalization was observed in the sarcoidosis case. These observations demonstrate that macular holes secondary to posterior uveitis frequently resolve without surgical intervention and so could be underdiagnosed if the patient is not evaluated at the time of onset before spontaneous hole closure.

## Review

### Introduction

Ocular inflammatory diseases can cause sight loss by macular impairment. Cystoid macular edema is the principal sight-threatening complication [1] in uveitis patients. Among possible macular inflammatory alterations, Nussenblatt describes macular hole occurrence as a very rare complication [2], being more often idiopathic or senile, postoperative or due to an epiretinal membrane, but rarely to posterior segment inflammation.

Here we report three cases of macular hole secondary to posterior uveitis that spontaneously resolved after etiological treatment, and present a review of relevant literature. Written informed consent was obtained from all patients for publication of this report.

#### Case no. 1

**Figure 1:**
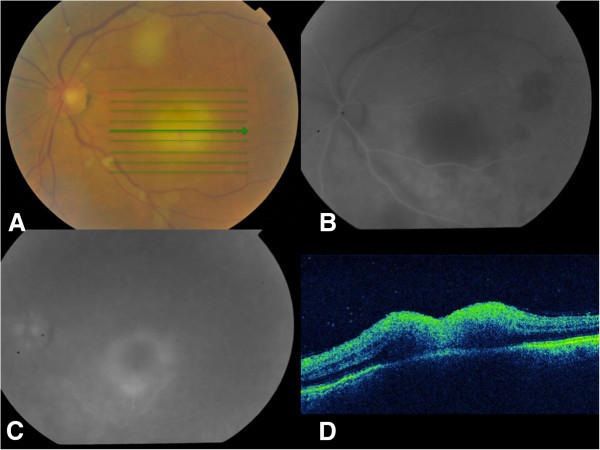
**Case 1, eye examination results before treatment. **(**A**) Retinophotography of the right eye: mild diffuse vitritis, vitreous aggregates (at the top of the macula and along the vessels), and macular chorioretinitis focus. (**B**, **C**) Fluorescein angiography of the right eye: centripetal impregnation of the macular lésion and temporal subclinical hypofluorescent area. (**D**) SD-OCT of the right eye: thickened hyper-reflective retina and inverted curvature of the posterior pole showing choroid impairment.

**Figure 2:**
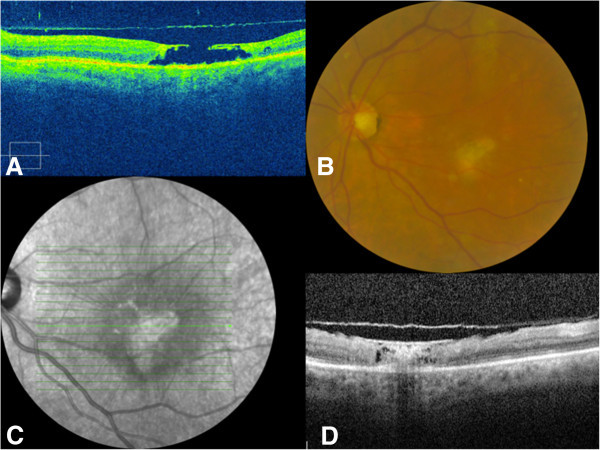
**Case 1, eye examination results after treatment. **(**A**) SD-OCT of the right eye: stage III macular hole. (**B**) Retinophotography of the right eye: a fibrous closure associated with an epiretinal membrane, spontaneously developed. (**C**) Red-free photography showing large macular fold due to fibrosis. (**D**) SD-OCT of the right eye: closure of the macular hole, filled with fibrosis.

A 58-year-old farmer presented for a sudden deterioration of visual acuity in his left eye. His visual acuity was 20/40 in his right eye and counting fingers in his left eye. Full examination of the right eye showed cataract. In the left eye, slit-lamp examination showed a slight Tyndall effect with small keratic precipitates. Ocular pressure was normal, and fundus examination showed a mild diffuse hyalitis with numerous vitreous aggregates (at the top of the macula and along the vessels) overlying a yellowish macular lesion estimated at two papillary diameters. It was not pigmented (Figure 1A). Fluorescein angiography showed a centripetal impregnation of the macular lesion (Figure 1B,C) as seen in toxoplasmosis infections [3], and a subclinical temporal hypofluorescent area in addition to the macular retinochoroidal lesion. Indocyanine green (ICG) angiography showed a late impregnation of the margins of the lesion. Spectral domain optical coherence tomography (SD-OCT) assessment showed a thickened hyper-reflective retina and an inverted curvature of the posterior pole, showing probable concomitant impairment of the choroid and confirming the diagnosis of chorioretinitis (Figure 1D). The Goldmann-Witmer coefficient was positive for *Toxocara canis*, and albendazole was given to the patient for 2 weeks. At the end of the treatment, the OCT displayed the appearance of a stage III macular hole (also evident at fundus examination), as the posterior hyaloid was detached from the fovea (Figure 2A). After a 6-month follow-up, the macular hole had spontaneously resolved, filled by fibrous scar tissue associated with an epiretinal membrane (Figure 2B,C,D). The final visual acuity in the left eye was 20/100 after a 3-year follow-up.

#### Case no. 2

**Figure 3:**
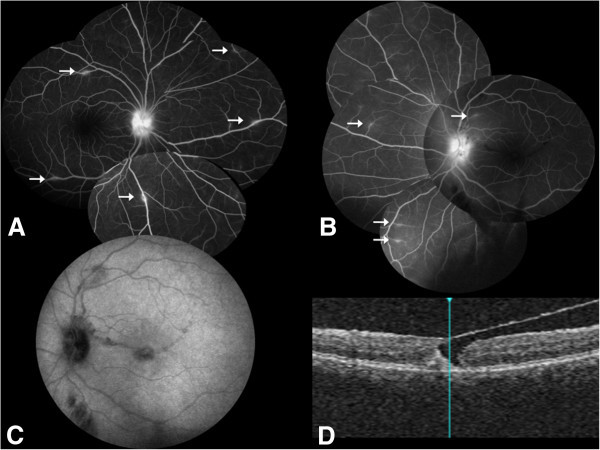
**Case 2, eye examination results before treatment. **(**A**, **B**) Fluorescein angiography of both eyes: bilateral optic disc edema and phlebitis (arrows). (**C**) Both fluorescein angiography and ICG angiography show hypofluorescent linear lesions in the macular area. (**D**) Macular SD-OCT of the left eye: pseudohole.

**Figure 4:**
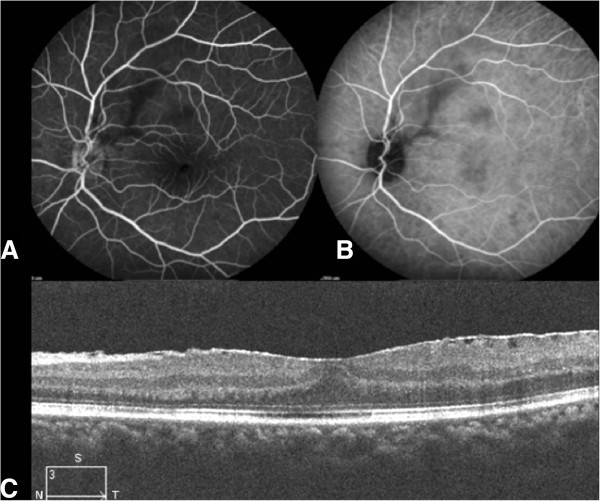
**Case 2, eye examination results after treatment. **(**A**, **B**) Fluorescein angiography of the left eye: healing of the optic disc edema and vascularitis. (**C**) Macular SD-OCT of the left fundus: closure of the macular hole promoted by the posterior hyaloid release and development of an epiretinal membrane.

A 28-year-old woman presented for sight loss in her left eye. Her visual acuity was 20/20 in her right eye and 20/100 in her left eye. Anterior chamber examination showed granulomatous keratic precipitates in the left eye but no Tyndall effect. Fundus examination showed bilateral optic disc edema and a macular hole on the left. Fluorescein angiography confirmed the bilateral optic nerve head diffusion and showed bilateral phlebitis (Figure 3A,B). Both fluorescein angiography and ICG angiography show hypofluorescent linear lesions in the macular area (Figure 3C). SD-OCT confirmed the presence of a pseudo macular hole (Figure 3D).The diagnosis workup was positive for sarcoidosis: increase in the angiotensin-converting enzyme to 160 U/L, mediastinal adenopathies on the computed tomography, increased rate of CD4/CD8 lymphocyte level in broncho-alveolar lavage, and multiple epithelioid and gigantocellular granuloma without caseous necrosis in analysis of bronchial biopsies. A prednisolone treatment at 1 mg/kg was given. After a 3-month follow-up, the visual acuity had increased to 20/25 in the left eye. Fundus examination and fluorescein angiography showed bilateral healing of the optic disc edema and vascularitis (Figure 4A,B). SD-OCT showed closure of the macular hole with normalization of the foveal photoreceptor layer and the development of an epiretinal membrane (Figure 4C). Examination remained stable after a 3-year follow-up.

#### Case no. 3

**Figure 5:**
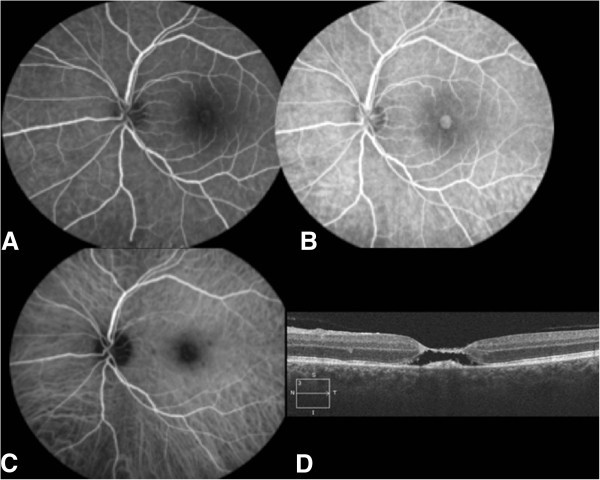
**Case 3, eye examination results before treatment. **(**A**, **B**) Fluorescein angiography of the left eye: macular window effect. (**C**) Indocyanine green angiography of the left eye: multiple hypofluorescent parafoveolar chorioretinitis foci. (**D**) Macular SD-OCT of the left eye: macular hole.

**Figure 6:**
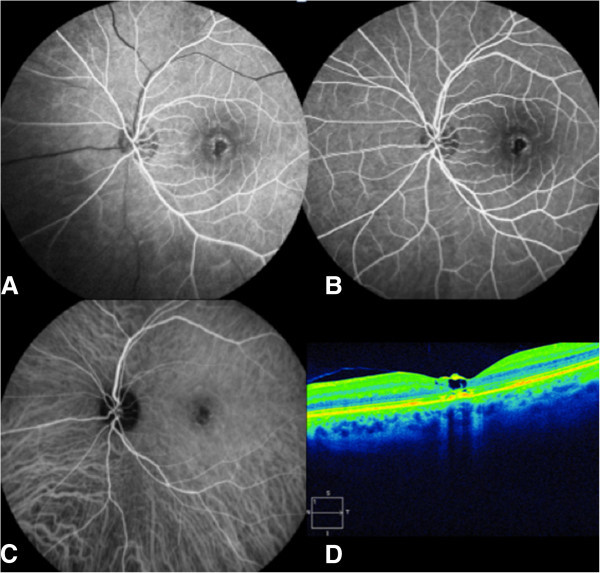
**Case 3, eye examination results after treatment. **(**A**, **B**) Fluorescein angiography of the left eye: macular RPE alterations. (**C**) Indocyanine green angiography of the left eye: healing of the chorioretinitis foci. (**D**) Macular SD-OCT of the left fundus: closure of hole with some cystoid alterations remaining.

A 39-year-old man was referred for a 2-day loss of visual acuity in his left eye. This patient had a medical history of hepatitis C infection, human immunodeficiency infection with normal T lymphocyte count and undetectable viral load, and a known evolutive syphilis infection. Being allergic to penicillin, he had been treated using oral tetracycline for several months with no decrease in serum *Treponema pallidum* hemagglutination assay-Venereal Disease Research Laboratory (TPHA-VDRL). His visual acuity was 20/20 in his right eye and counting fingers in his left eye. Slit-lamp examination showed a mild Tyndall effect in the anterior chamber and a macular hole (Figure 5A,B). Fluorescein angiography showed the macular hole; indocyanine green angiography revealed multiple hypofluorescent parafoveolar chorioretinitis foci (Figure 5C), and SD-OCT confirmed the hole (Figure 5D). A complete inflammatory and infectious assessment found only increased serum TPHA-VDRL. The patient was treated by intravenous ceftriaxone at 2 g/day for 1 week and then at 1 g/day for a further week. One month after treatment, the angiography showed healing of the chorioretinitis foci (Figure 6A,B,C). After a 2-year follow-up, visual acuity had increased to 20/40, and the OCT showed a subtotal closure of the macular hole with some cystoid alterations remaining (Figure 6D).

**Figure 7:**
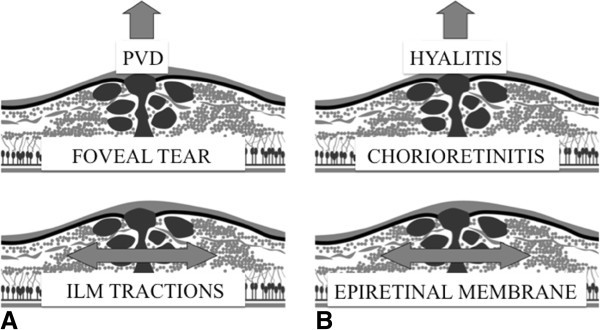
Physiopathology of (A) iodiopathic macular holes compared with (B) macular holes in circumstances of inflammation.

### Discussion

Intraocular inflammatory diseases have long been described as a major cause of blindness [1]. In all kinds of uveitis, bilateral legal blindness develops in 4% of patients, unilateral blindness in 14%, bilateral visual impairment in 6%, unilateral impairment in 11%, and unilateral impairment with blindness of the other eye in 4% to 5%.

The occurrence of a macular hole in a context of uveitis is an unusual, little-described complication.

In 1986, Nussenblatt [2] studying macular alterations in uveitic patients described this evolution as ‘uncommon’. Later, in a retrospective study of 582 patients with uveitis [1], no macular hole was described. Neither prevalence nor incidence of macular holes linked to posterior segment inflammation can be established from the literature, cases or short series being too scant.

On the other hand, in a histopathological study of 17 lamellar and 18 full-thickness macular holes [4], mild to moderate choroiditis was present in six eyes in the macular area. One of these cases was suspected of being an incidental finding because the choriocapillaris, Bruch's membrane, and the retinal pigment epithelium (RPE) were intact. This study gives us an approximate figure for the proportion of macular holes linked to posterior uveitis in all macular holes. However, there is a selection bias: the eyes of this study were obtained from enucleations or post-mortem, so the results do not reflect the general epidemiology of the disease.

The literature reports 55 cases of macular hole secondary to posterior uveitis (Table 1): 35 Behcet's disease [5-11], 3 idiopathic intermediate uveitis [9], 2 idiopathic posterior uveitis [9], 1 bilateral intermediate uveitis [12], 2 *Bartonella henselae*[13,14], 2 toxoplasmosis [15,16], 2 Vogt-Koyanagi-Harada [17], 1 cytomegalovirus (CMV) retinitis [9], 1 HLA B27 uveitis [18], 1 bilateral anterior uveitis [19], 1 serpiginous choroiditis [20], 1 fungal endophthalmitis [21], 1 T/natural killer (NK) lymphoma [22], 1 presumed histoplasmosis [23], and 1 immune recovery uveitis in an AIDS patient after CMV retinitis [24].

**Table 1:** Review of the literature of macular holes in a context of intraocular inflammation

**Etiology**	**Number of cases**	**Study**
Behcet's disease	35	[5-11]
Idiopathic intermediate uveitis	4	[9,12]
Idiopathic posterior uveitis	2	[9]
*Bartonella henselae*	2	[13,14]
Toxoplasmosis	2	[15,16]
Vogt-Koyanagi-Harada	2	[17]
CMV retinitis	1	[9]
HLA B27	1	[18]
Bilateral idiopathic anterior uveitis of unknown etiology	1	[19]
Serpiginous choroiditis	1	[20]
Fungal endophthalmitis	1	[21]
T/NK lymphoma	1	[22]
Presumed histoplasmosis	1	[23]
Immune recovery uveitis in AIDS patients with CMV retinitis	1	[24]

Idiopathic macular holes can be explained by a triple constraint [25]: (1) an antero-posterior traction, often effected by the posterior vitreous detachment; (2) a tangential traction especially when there is an epiretinal membrane or a lateral shifting of vitreoretinal adherence; and (3) an intraretinal constraint that weakens the inner retinal surface by inducing cystic changes and contributes to the macular hole (Figure 7A). A parallel can be done in circumstances of posterior segment inflammation: the inflammation often induces changes or liquefaction of the vitreous body and contributes to the posterior vitreous detachment which realizes the first constraint. The development of an epiretinal membrane is classical in uveitis [26], responsible of a tangential traction, as seen in the cases 1 and 2. Finally, chorioretinitis foci or cystoid macular edema cause the third constraint, fragilizing the retinal tissue (Figure 7B). This explains why treating the inflammation can relieve these constraints or tractions and produce spontaneous healing of the macular hole. The report of Halkiadakis et al. [18] on a spontaneous macular hole closure after peribulbar steroid injection in the case of a HLA B27 uveitis demonstrates this well.

The relatively old references on the low prevalence of macular hole in uveitic patients could be explained by the fact that OCT is a recent tool. Our cases also show the importance of the SD-OCT in uveitis to confirm cystoid macular edema, the principal sight-threatening effect in uveitis [1]. OCT can also be a diagnostic criterion in some etiologies such as intermediate uveitis or a poor-prognosis marker as in Birdshot retinochoroidopathy, indicating a specific care requirement. OCT is necessary during follow-up after resolution of active inflammation to seek the cause of any unexplained sight loss and may reveal a closed macular hole filled by fibrosis.

SD-OCT is useful for the preoperative evaluation of the inflammatory secondary epiretinal membrane to evaluate the benefits of the procedure: in our first case, in which an epiretinal membrane developed, there was a partial posterior vitreous detachment, and so there is a persistent risk of macular hole recurrence in a such damaged and weakened retina, with very small hope of visual improvement.

## Conclusion

The occurrence of a macular hole linked to posterior uveitis is an uncommon but possibly underestimated cause of sight loss. Etiological treatment seems to lead to macular hole closure in most such cases. SD-OCT is very useful for assessing the evolution of these cases and should be performed whenever there is a posterior involvement or unexplained sight loss in uveitis patients.

## Competing interest

The authors declare that they have no competing interests.

## Authors’ contributions

NB wrote the manuscript, synthetized the cases, and made the literature review. PLC was the co-author; he corrected and directed the redaction of the manuscript and followed the cases. FC, EL, HJM, AF, and LP participated to the management of the cases and the diagnosis. FC, FB, and CB participated in the design of the study and its coordination. All authors read and approved the final manuscript.
